# The Effect of Symmetric and Asymmetric Loading of Frontal Segment with Two Curved Cantilevers: An In Vitro Study

**DOI:** 10.3390/dj10040052

**Published:** 2022-03-29

**Authors:** Malgorzata Bilinska, Michel Dalstra

**Affiliations:** Section of Orthodontics, Department of Dentistry and Oral Health, Aarhus University, Vennelyst Boulevard 9, 8000 Aarhus, Denmark; michel.dalstra@dent.au.dk

**Keywords:** cantilevers, dental asymmetry, midline correction, three-piece intrusion arch

## Abstract

Cantilevers generate statically determined force systems. The frontal segment loading with symmetric and asymmetric cantilevers in a three-piece intrusion base arch can be used to correct midline asymmetry. Three types of 0.017″ × 0.025″ beta-titanium cantilevers: tip-back (TB), deep curve (DC), utility arch (UA) were tested on typodonts simulating intrusion of the maxillary anterior segment. Typodonts with symmetric and asymmetric cantilevers were scanned with intraoral scanner (3Shape, TRIOS, Copenhagen, Denmark) before (T0) and after (T1) the experiment, scans were superimposed using Mimics software (Materialise, Leuven, Belgium). Data were analysed with qualitative analysis. All cantilevers generated vertical and horizontal forces. For symmetric design, the DC and TB displayed intrusive force with retrusive component, UA intrusion and protrusion. The asymmetric cantilevers produced transverse displacement of anterior segment. DC created lateral, UA medial force, the anterior segment displacement was consistent with the used configuration. The movement of an anterior segment with TB is smaller compared to DC and UA. Symmetric cantilevers configurations can achieve simultaneous intrusion and retrusion or protrusion of the anterior segment. The asymmetric design with transversal force can clinically aid the correction of midline discrepancies. The effect of the cantilever configuration on delivered force direction was confirmed.

## 1. Introduction

Cantilevers produce statically determined force systems. When activated, a force is generated as one-point contact, while in the reactive unit a moment and force with opposite directions occur [1]. Force application is limited to an area and its magnitude is low due to a long inter-attachment distance and the low deflection rate of a beta titanium wire (TMA). The observed side effects are minimal [2]. Using a simple design, powerful biomechanical force systems can be applied to move teeth according to the plan of orthodontic treatment. Numerous one-couple appliances are described in the literature designed to move a group of teeth or an individual tooth in all three dimensions of space [3]. Cantilevers are useful in solving multiple clinical issues, including molar uprighting [4,5], correction of dental asymmetry [6], extrusion of impacted canines [7,8,9,10,11], correction of vertical discrepancies by intrusion of a single tooth [12] and correction of the canted occlusal plane [13,14]. Cantilevers can also be used to correct a dental or alveolar asymmetry or camouflage minor skeletal asymmetry by improving the midline deviation [15]. Cantilevers can provide a pulling force, which is capable of shifting the displaced midline to the desired position [16]. For correction of deep bite, a three-piece intrusion arch may be the appliance of choice. It consists of one front segment with two active cantilever springs on each side. The created force system results in anterior segment intrusion, and at the same time posterior extrusion and molar tip back occur [16]. The line of action passes close to the centre of resistance. The tip-back mechanics will provide a vertical force. An additional horizontal force component generates the line of action parallel to the long axis of the tooth, which results in retraction of the proclined teeth [17].

According to the literature, the estimated force required for intrusion of the upper front segment varies between 60 g and 150 g [18,19,20]. Cantilever design influences the force system: its configuration has an effect on the magnitude and direction of delivered force. In a two-dimensional model with finite element analysis (FEA), Dalstra and Melsen presented evidence that curved cantilevers directly generate a retrusive force, while utility-arch formed ones generate an initial protrusive force. The force systems perform in a three-dimensional setting; therefore, cantilever configuration may be used for specific clinical indications [1]. The treatment plan requires careful diagnosis and proper biomechanical planning in order to achieve predictable results [2]. According to our knowledge, no study has been previously published analysing the different effects of direct tooth movement with the use of symmetric and asymmetric cantilever configurations. The aim of the study is to carry out a qualitative analysis and observation of tooth movement as the effect of loading the frontal segment with symmetric and asymmetric cantilevers in an intrusion three-piece base arch. The study hypothesis was that activation of symmetric cantilevers will result in symmetric tooth movement and the effect of asymmetric cantilever design will result in asymmetric displacement of the anterior segment.

## 2. Materials and Methods

**Figure 1:**
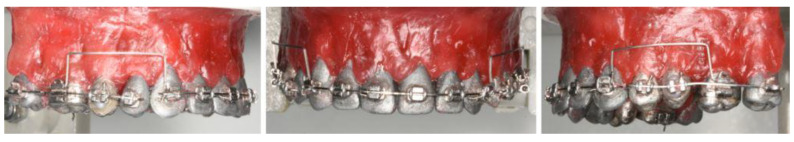
Symmetric activation of 10 mm UA (typodont).

**Figure 2:**
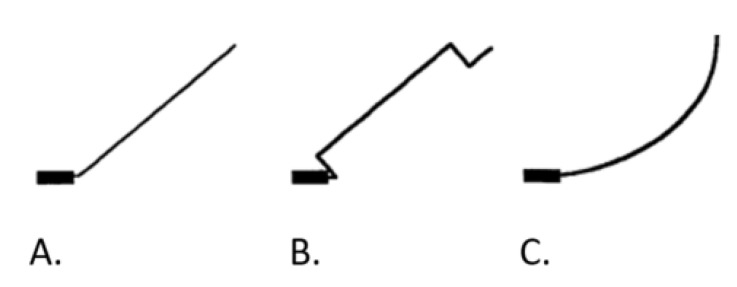
Cantilever design: (**A**). tip-back (TB) (**B**). utility arch (UA) (**C**). deep curve (DC).

The cantilevers were tested on a typodont (wax) simulating intrusion of the anterior segment in the upper arch with a design analogous to the classic three-piece intrusion arch [19,21]. The anterior segment consisted of a 0.018″ × 0.025″ stainless steel (SS) arch wire, sectioned between the canines and first premolars on both sides. Maxillary incisors and canines were included. A 0.018″ × 0.025″ SS side segments and transpalatal arch (TPA), fabricated from 0.08″ stainless steel wire, was inserted into sheets of the first molars bands to reinforce the anchorage (Figure 1). The cantilever designs used in the current study originate from Dalstra and Melsen (1999). Three types were chosen: tip-back (TB), deep curve (DC) and a 6 mm high utility arch (UA) [1] bent from 0.017″ × 0.025″ beta-titanium (TMA) wire (Figure 2). The cantilevers were activated upwards on both sides and attached from the first molar auxiliary tube to the sectioned anterior arch wire between the lateral incisor and canine. This point of attachment was selected to obtain an intrusive force. The amount of activation was measured as a distance from the activated cantilever to the point of ligation located on the anterior segment. The study consisted of two parts: In the first part, the symmetric cantilever design was placed bilaterally: DC, TB and UA. All tests were repeated twice to assess the reproducibility of the results. The cantilevers were activated symmetrically: the activation range was 10 mm. In the second part, asymmetric activation was applied: different cantilever designs were located on the right and left side: DC/UA, DC/TB, TB/DC, TB/UA, UA/DC and UA/TB. All cantilevers were activated at 10 mm. After cantilevers activation and ligation, the typodonts were placed in a water bath at a temperature of 60 degrees Celsius for 5 min, then cooled down in cold water to ensure the hardening of the wax. The drop in water temperature after typodont placement was controlled with an electronic thermometer. The typodonts were scanned using an intraoral scanner (3Shape, TRIOS, A/S, Copenhagen, Denmark); both before the cantilever placement (T0) and after the experiment was performed and wax had cooled down (T1). The obtained T0- and T1-scans were superimposed using Mimics software (Materialise, Leuven, Belgium). Data were analysed with qualitative analysis. All tests were repeated two times to assess the repeatability.

## 3. Results

During deactivation, all studied cantilevers presented vertical and horizontal forces. In a vertical direction intrusion was observed for all symmetric and asymmetric configurations. The assessment of the type of movement was performed on superimposed scans separately for all six maxillary teeth.

**Figure 3:**
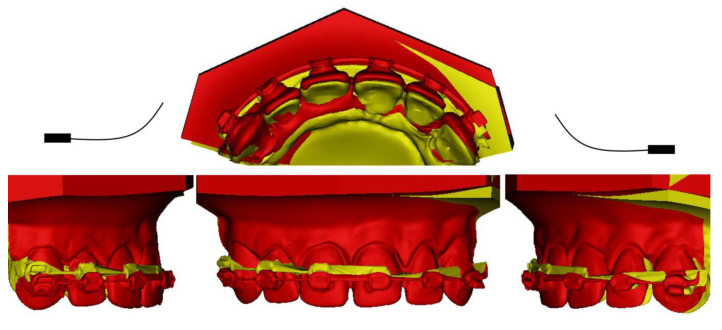
Symmetric activation of 10 mm DC (T0: red, T1: yellow).

For symmetric cantilevers, the DC configuration displayed an intrusive force with a slight retrusive component. Similar results were found for the TB. For the UA, intrusion and protrusion was observed. The results are summarized in Table 1. The orthodontic simulations on the typodont models—the superimposition of the T0 and T1 models for symmetric design—are shown in Figure 3.

**Figure 4:**
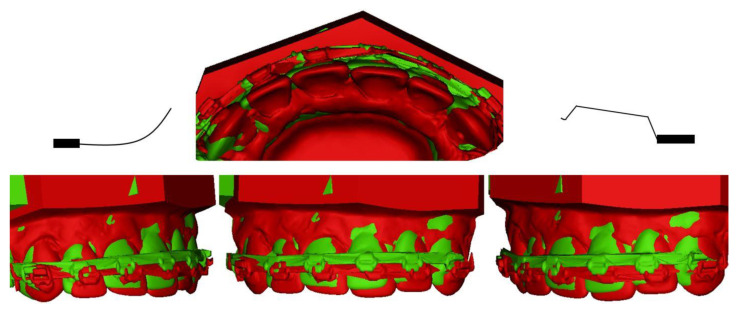
Asymmetric activation of 10 mm DCUA (T0: red, T1: green).

In the asymmetric designs, the results were analysed in the vertical and horizontal directions where sagittal movement of the front segment was investigated. The resultant lateral movement was analysed in the transversal plane. The asymmetric placement of cantilevers produced dental movement with transversal displacement of anterior segment. Tip back cantilevers produced less movement in the transverse plane, compared to DC and UA. DC created a lateral force, while UA a medial force. The displacement of the anterior segment was observed with movement according to the used configuration (Figure 4). With the application of DC, the lateral movement was observed toward the DC-side, both with TB and AU cantilevers on the contralateral side. When UA was applied together with TB, the displacement was less pronounced. The same observation was made for DC and TB: the lateral movement of the anterior segment was smaller compared to DC and UA. The results are summarized in Table 2. Both experiments for symmetric and asymmetric cantilevers have shown excellent repeatability with insignificant differences. The analysis of the anchorage unit revealed no significant movement of molars.

## 4. Discussion

The study hypothesis was confirmed: activation of symmetric cantilevers results in symmetric movement of anterior segment and the asymmetric cantilever design produces asymmetric movement.

Segmented cantilever mechanics creates a statically determined force system and therefore the generated tooth movement is predictable. TMA has a low load-deflection rate and offers a large range of activations with less frequent reactivations needed. Undesired forces and moments are transferred to the anchorage unit, which allows the control of the side effects [15,22]. All experiments were carried by the same operator on the same day; therefore, other external influences were minimalized. Wires used in the current study originated from the same package with the same reference number to avoid variation between batches. In the current study, all cantilevers were bent manually: despite precision and care, small differences may have occurred. Even though all typodonts came from same manufacturer, small differences were observed in tooth movement may be explained by uneven wax thickness around the teeth. A limited number of typodonts were placed in the bath at once; the controlled water temperature drop was insignificant and had no impact on typodont tooth movement in the wax. Moreover, the warm wax is very sensitive for distortions, so the cantilevers were not removed before complete cool down. The use of an intraoral scanner ensured precise scans and it was possible to perform precise superimposition. The superimposition was useful to perform analysis of tooth movement and was a convenient tool to visualize the changes in tooth position. In the symmetric cantilever placement, the retraction was observed for DC and TB, and proclination for UA. The results are consistent with study by Dalstra and Melsen: the curved cantilevers displayed a varying amount of retraction throughout the deactivation, while TB and UA exhibited similar deformation patterns with protrusion force, with retrusive component after deactivation [1]. In our study, the TB design was fitted to the arch shape, so it could be attached to the front segment, while in the study by Dalstra et al., it was straight. The curvature may explain the retrusive force delivered by both DC and TB designs. According to Dalstra and Melsen, the turning point between protrusion and retraction forces depended on the amount of activation. In our study, all cantilevers were activated for 10 mm activation range. Study by Dalstra and Melsen was performed as a laboratory experiment with the use of finite element models, while the current study was performed on typodonts [1]. On the other hand, the small differences between repeated experiments may be associated with the error of the method due to the features of the typodonts.

### 4.1. Clinical Implications

The aim of the current study was to accomplish new insights in the mechanics of orthodontic cantilevers, and how these can be applied in a clinical setting. Clinically, a true intrusion of the incisors is rarely desired. More frequently, a combination of intrusion and either protrusion or retraction is needed to solve the deep bite malocclusion [17,22]. Different designs of the cantilever configuration could be chosen to obtain the desired result. Since the utility-shaped configurations produced a protrusive force, for an intrusion combined with a simultaneous protrusion, a utility-shaped configuration should be applied. Similarly, when intrusion combined with a simultaneous retraction of the front teeth segment is desired, then curved cantilever and tip-back configurations are capable of producing a retrusive force [1]. In a study by van Steenbergen et al., 40 g and 80 g activation for maxillary anterior segment were applied. The authors concluded that there was no statistically significant difference between both activations in rate of intrusion of incisors, amount of axial inclination and effect on buccal segments [20]. The side effects on the buccal segments were not addressed in the present study, but the use of a TPA was applied to minimalize the molar movements. The study by van Steenbergen et al. was performed clinically on patients, therefore the effect of time of application of cantilever activation, occlusal forces and bone biology of dental intrusion was a factor, which was not taken into consideration in the current study. Theoretically, the effect of the use of higher forces will lead to the presence of more pronounced side effects, but also may produce an increased rate of root resorption, as the most damage is observed with intrusive mechanics as this concentrates pressure at the apex of a tooth [20,23]. According to Shroff et al., the successful intrusion and retraction of incisors with a segmented approach delivers a constant and low level of force. A well-controlled statically-determined force system requires minimal chairside adjustments [22].

Despite upper segment intrusion, the setting with two cantilevers and an anterior segment can be used to correct upper midline discrepancies, as described by Bergamini and Melsen [6]. As the asymmetric cantilever design is known to produce buccal force, it could aid to the shift the upper segment and achieve coinciding midlines. Nanda and Margolis stated that cantilevers can be used for changing the axial inclinations in patients with apical base discrepancies. The use of low forces with minimal side effects favour the use of cantilevers for uprighting tipped incisors and the correction of deviated midline [2]. According to Kuhlberg et al., adding a passive loop apically toward the centre of resistance of the anterior teeth will produce bodily movement, as the force is applied at the desired level: close to the centre of resistance [16]. On the contrary, Lindauer et al. proposed a simultaneous midline correction on a full wire setting; they described the use of a single cantilever, which was tied to the full arch wire. In the current study, the side effect on the molars was minimal and insignificant. However, in the clinical setting, cantilevers reactivation, as well as the treatment time, delivers force over a much longer time and the use of proper anchorage unit is important. As the cantilever is activated, a second-order couple developed in the molar to rotate it mesiolingually, so the anchorage should be carefully assessed [3]. The use of anchorage is strongly advised to help to minimalize the side effects. Bergamini and Melsen concluded that treatment of dental asymmetries may be difficult, because the side effect of using a biomechanical approach may create new symptoms of malocclusion [6]. To avoid side effects of molar rotations and distal tipping, it is important to unite the posterior units with a transpalatal arch, connecting the molars. The anchorage units should be carefully monitored and, if needed, activated to counter the forces generated by the cantilevers [15]. As the transpalatal arch may still allow some degree of distal tipping of upper molars, DeVincenzo and Winn suggested the use of a Nance appliance with intrusion arches, where the soft tissue resistance provides more stability [24].The use of mini-implants aids the achievement of absolute anchorage: mini-implants eliminate the side effects of conventional treatment methods and may diminish the need for complicated mechanics [25].Albelasy et al. proposed the simultaneous intrusion and retraction of maxillary central and lateral incisors using a mini-implant supported three-piece Burstone base arch [26]. A mini screw-supported Burstone intrusion arch offers the advantage of true incisor intrusion without causing reciprocal effects on the posterior teeth [27]. In the study by Aras et al., Burstone’s three-piece intrusion arch was modified with TMA cantilevers attached directly to the mini-implants. According to authors, this design can reduce the amount of risk resorption, compared to the direct traction to the mini screws in the anterior region. When the initial incisors are upright, labial flaring is desired [25]. Comparing direct intrusion to the mini screws, El Namrawy et al. established that there was no difference in the extent of maxillary incisor intrusion with mini screws and intrusion arch, beside the presence of incisor proclination in the intrusion arch group [28]

### 4.2. Limitations of the Present Study

The obtained results are comparable and presented a consistent behaviour of the material as it is a preliminary in vitro study on a typodont. The present study should only be viewed and considered as a proof of concept, because there are still several limitations to concern. A typodont consists of artificial teeth embedded in wax, which substitutes the periodontium. In a clinical setting, bone modelling, occurring when orthodontic forces are applied, is a result of complex biomechanical reactions in biological tissues of the periodontium [29]. The observed movement of teeth may vary from the expected teeth movement in the mouth: there are no occlusal forces, which may have an influence on the final teeth position, also the impact of the temperature change from hot food and drinks was neglected in the clinical situation [30]. The study could not be performed clinically: the use of cantilevers and an undesired result would lead to round-tripping and therefore a higher risk of root resorption [26]. Moreover, it would not be possible to perform randomization due to specific treatment needs. Another point of consideration is the different bone metabolism among the patients and variable speed of teeth movement. The obtained results would not be precise. The use of typodonts allows us to perform simulations of different settings of anterior segment movement.

## 5. Conclusions

The results from the present study show that symmetric cantilever configurations achieve simultaneous intrusion and retrusion or protrusion of the anterior segment. In some clinical settings, asymmetric cantilever designs can clinically aid the correction of midline discrepancies as a asymmetric transversal force is generated. The effect of the cantilever configuration on the delivered force direction was thus confirmed.

## Figures and Tables

**Table 1:** Symmetric activation of cantilevers (R: retrusion, P: protrusion, I: intrusion).

Cantilever/Plane	Sagittal	Vertical
DC	R	I
TB	R	I
UA	P	I

**Table 2:** Asymmetric activation of cantilevers (R—retrusion, P—protrusion, I—intrusion, M—medial displacement, L—lateral displacement, 0—no change).

	Sagittal					
	13	12	11	21	22	23
DCUA	R	R	0	P	P	P
DCTB	R	R	R	0	R	R
TBDC	R	R	R	R	R	R
TBUA	R	R	0	0	R	R
UADC	P	P	P	0	0	R
UATB	P	P	P	P	0	R
	Transversal					
	13	12	11	21	22	23
DCUA	L	L	0	M	M	M
DCTB	L	L	L	0	M	M
TBDC	0	0	M	L	L	L
TBUA	0	0	0	M	M	M
UADC	M	M	M	L	L	L
UATB	M	M	M	L	0	0
	Vertical					
	13	12	11	21	22	23
DCUA	I	I	I	I	I	I
DCTB	I	I	I	I	I	I
TBDC	I	I	I	I	I	I
TBUA	I	I	I	I	I	I
UADC	I	I	I	I	I	I
UATB	I	I	I	I	I	I

## Data Availability

Not applicable.
